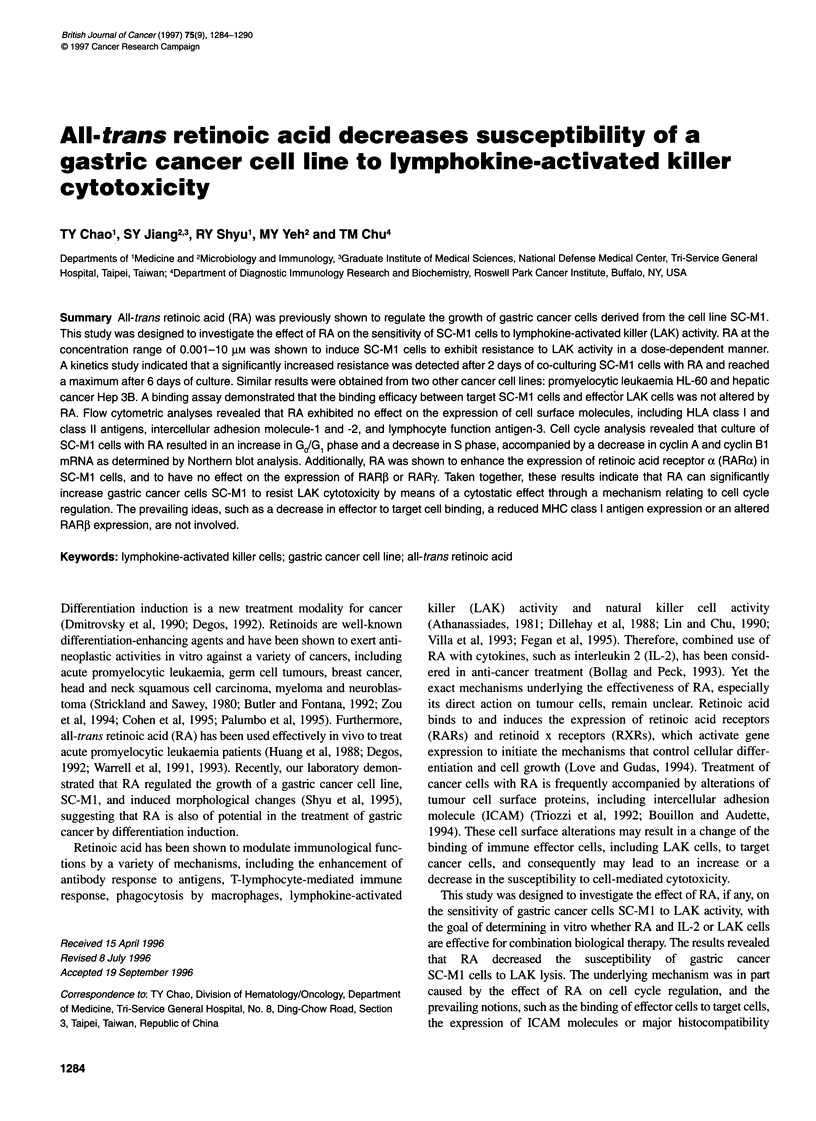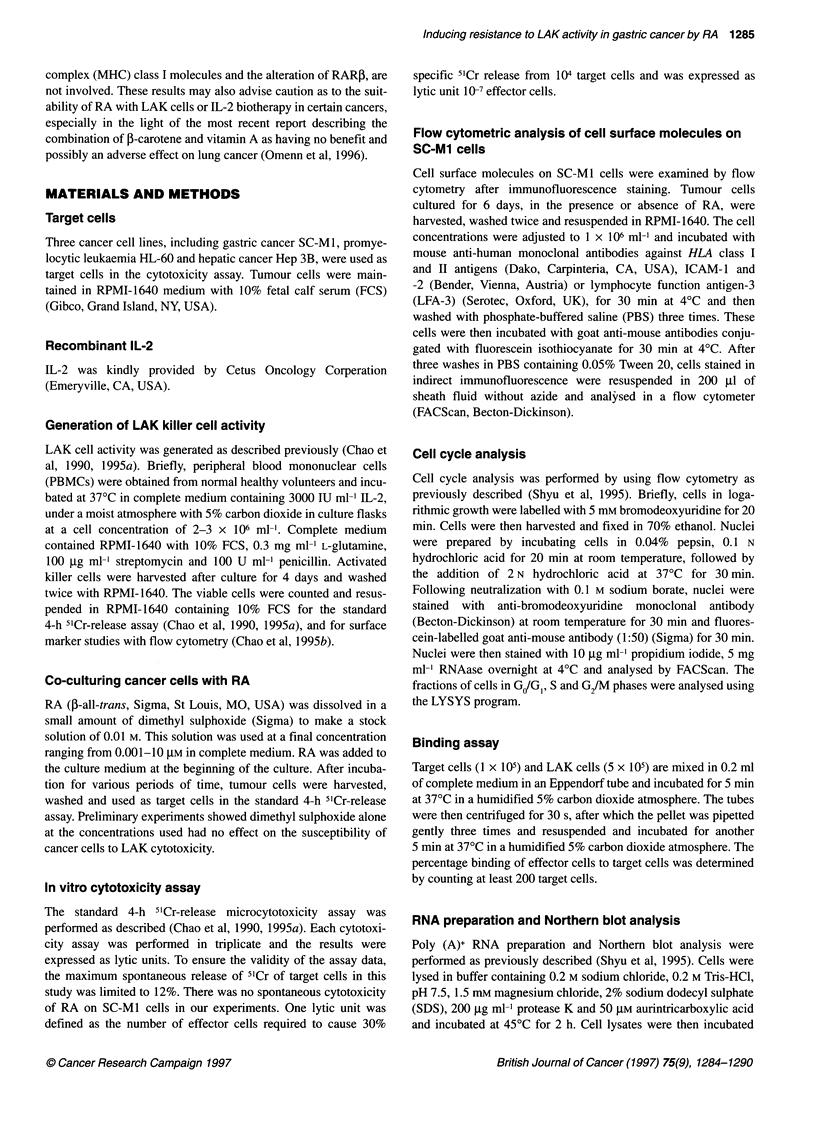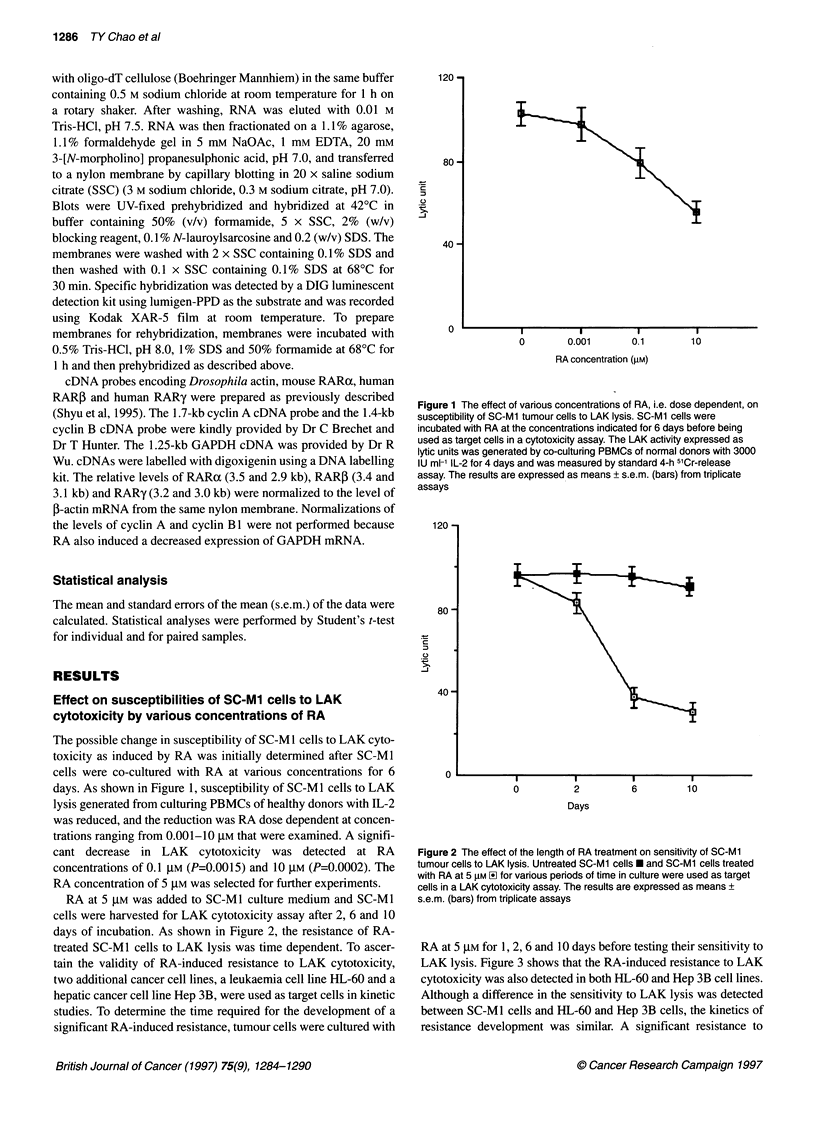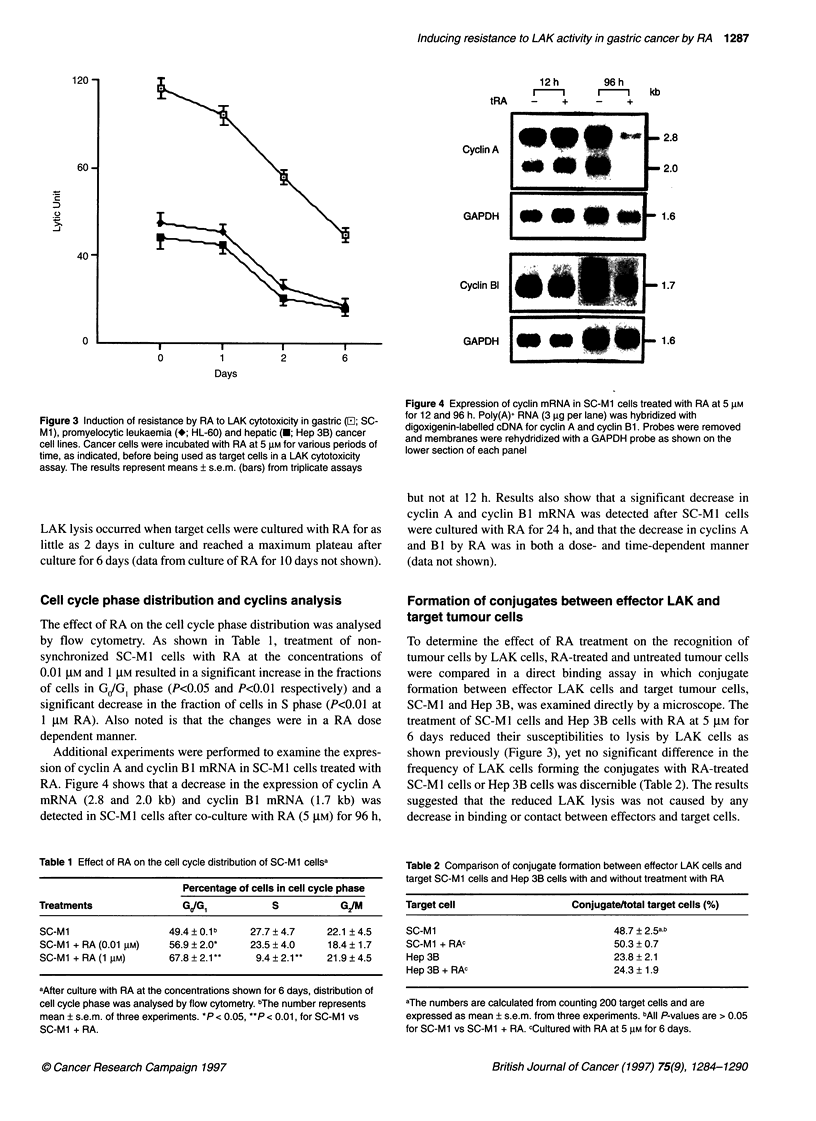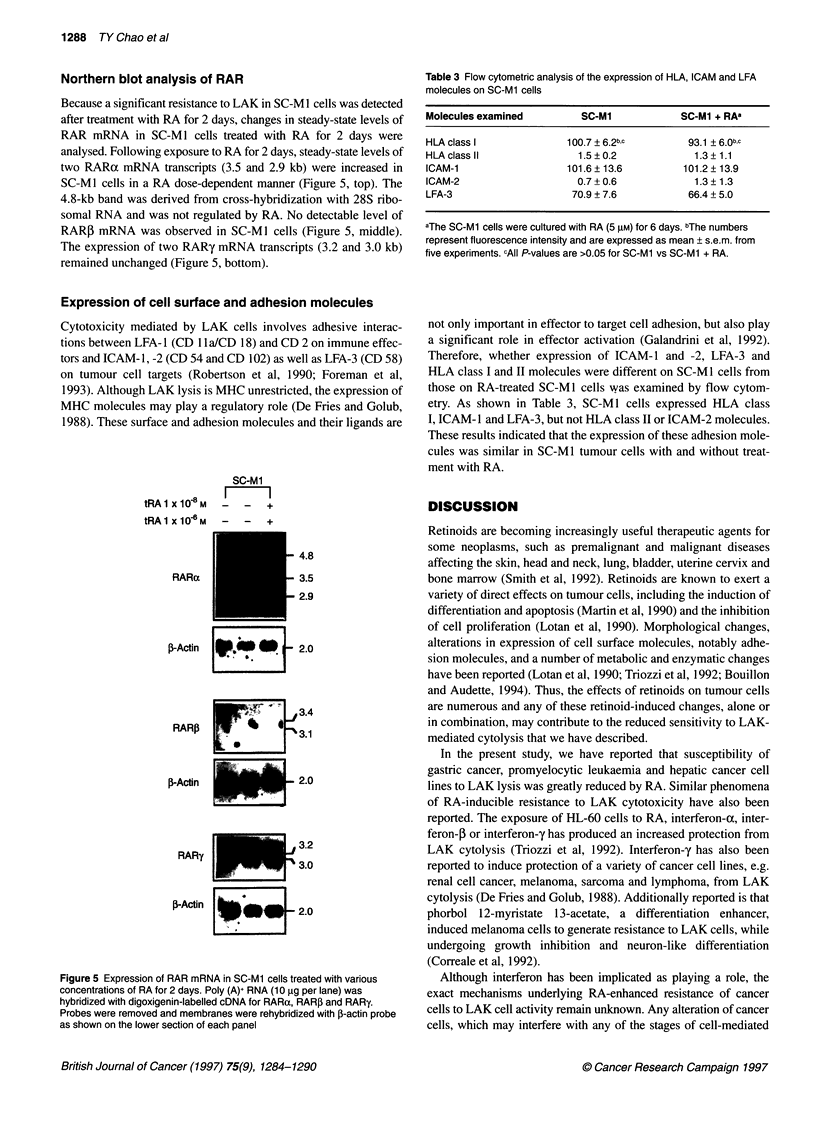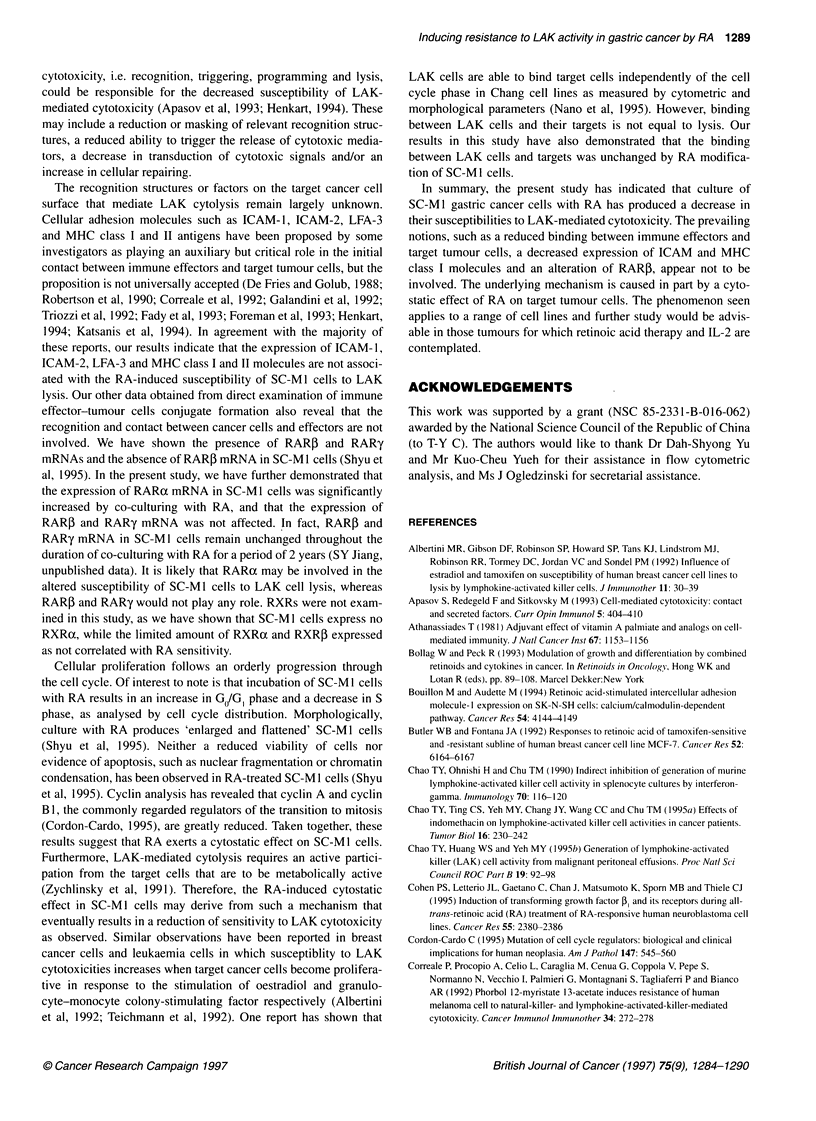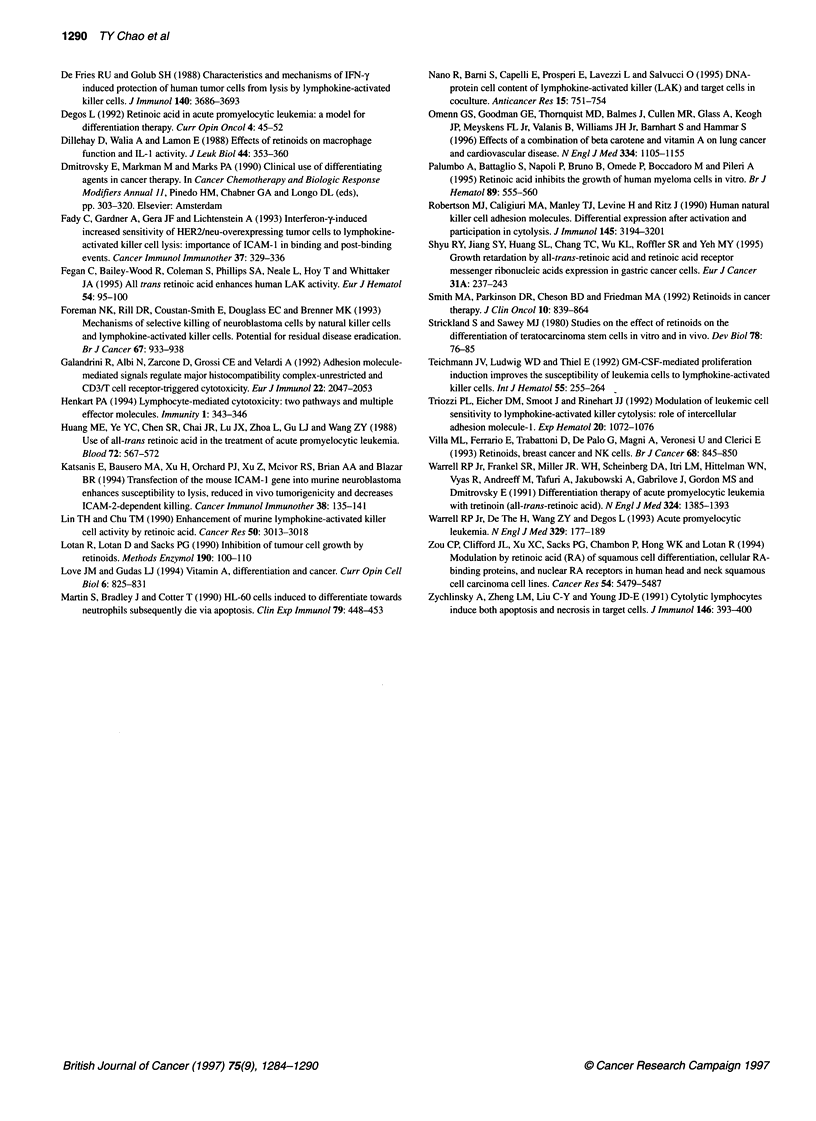# All-trans retinoic acid decreases susceptibility of a gastric cancer cell line to lymphokine-activated killer cytotoxicity.

**DOI:** 10.1038/bjc.1997.218

**Published:** 1997

**Authors:** T. Y. Chao, S. Y. Jiang, R. Y. Shyu, M. Y. Yeh, T. M. Chu

**Affiliations:** Department of Medicine, Tri-Service General Hospital, Taipei, Taiwan, Republic of China.

## Abstract

**Images:**


British Journal of Cancer (1997) 75(9), 1284–1290
© 1997 Cancer Research Campaign

### All-*trans* retinoic acid decreases susceptibility of a gastric cancer cell line to lymphokine-activated killer cytotoxicity

**TY Chao[1], SY Jiang[2,3], RY Shyu[1], MY Yeh[2] and TM Chu[4]**

Departments of [1]Medicine and [2]Microbiology and Immunology, [3]Graduate Institute of Medical Sciences, National Defense Medical Center, Tri-Service General Hospital, Taipei, Taiwan; [4]Department of Diagnostic Immunology Research and Biochemistry, Roswell Park Cancer Institute, Buffalo, NY, USA

**Summary** All-*trans* retinoic acid (RA) was previously shown to regulate the growth of gastric cancer cells derived from the cell line SC-M1. This study was designed to investigate the effect of RA on the sensitivity of SC-M1 cells to lymphokine-activated killer (LAK) activity. RA at the concentration range of 0.001–10 μM was shown to induce SC-M1 cells to exhibit resistance to LAK activity in a dose-dependent manner. A kinetics study indicated that a significantly increased resistance was detected after 2 days of co-culturing SC-M1 cells with RA and reached a maximum after 6 days of culture. Similar results were obtained from two other cancer cell lines: promyelocytic leukaemia HL-60 and hepatic cancer Hep 3B. A binding assay demonstrated that the binding efficacy between target SC-M1 cells and effector LAK cells was not altered by RA. Flow cytometric analyses revealed that RA exhibited no effect on the expression of cell surface molecules, including HLA class I and class II antigens, intercellular adhesion molecule-1 and -2, and lymphocyte function antigen-3. Cell cycle analysis revealed that culture of SC-M1 cells with RA resulted in an increase in $G_0/G_1$ phase and a decrease in S phase, accompanied by a decrease in cyclin A and cyclin B1 mRNA as determined by Northern blot analysis. Additionally, RA was shown to enhance the expression of retinoic acid receptor α (RARα) in SC-M1 cells, and to have no effect on the expression of RARβ or RARγ. Taken together, these results indicate that RA can significantly increase gastric cancer cells SC-M1 to resist LAK cytotoxicity by means of a cytostatic effect through a mechanism relating to cell cycle regulation. The prevailing ideas, such as a decrease in effector to target cell binding, a reduced MHC class I antigen expression or an altered RARβ expression, are not involved.

**Keywords:** lymphokine-activated killer cells; gastric cancer cell line; all-*trans* retinoic acid

Differentiation induction is a new treatment modality for cancer (Dmitrovsky et al, 1990; Degos, 1992). Retinoids are well-known differentiation-enhancing agents and have been shown to exert anti-neoplastic activities in vitro against a variety of cancers, including acute promyelocytic leukaemia, germ cell tumours, breast cancer, head and neck squamous cell carcinoma, myeloma and neuroblastoma (Strickland and Sawey, 1980; Butler and Fontana, 1992; Zou et al, 1994; Cohen et al, 1995; Palumbo et al, 1995). Furthermore, all-*trans* retinoic acid (RA) has been used effectively in vivo to treat acute promyelocytic leukaemia patients (Huang et al, 1988; Degos, 1992; Warrell et al, 1991, 1993). Recently, our laboratory demonstrated that RA regulated the growth of a gastric cancer cell line, SC-M1, and induced morphological changes (Shyu et al, 1995), suggesting that RA is also of potential in the treatment of gastric cancer by differentiation induction.

Retinoic acid has been shown to modulate immunological functions by a variety of mechanisms, including the enhancement of antibody response to antigens, T-lymphocyte-mediated immune response, phagocytosis by macrophages, lymphokine-activated killer (LAK) activity and natural killer cell activity (Athanassiades, 1981; Dillehay et al, 1988; Lin and Chu, 1990; Villa et al, 1993; Fegan et al, 1995). Therefore, combined use of RA with cytokines, such as interleukin 2 (IL-2), has been considered in anti-cancer treatment (Bollag and Peck, 1993). Yet the exact mechanisms underlying the effectiveness of RA, especially its direct action on tumour cells, remain unclear. Retinoic acid binds to and induces the expression of retinoic acid receptors (RARs) and retinoid x receptors (RXRs), which activate gene expression to initiate the mechanisms that control cellular differentiation and cell growth (Love and Gudas, 1994). Treatment of cancer cells with RA is frequently accompanied by alterations of tumour cell surface proteins, including intercellular adhesion molecule (ICAM) (Triozzi et al, 1992; Bouillon and Audette, 1994). These cell surface alterations may result in a change of the binding of immune effector cells, including LAK cells, to target cancer cells, and consequently may lead to an increase or a decrease in the susceptibility to cell-mediated cytotoxicity.

This study was designed to investigate the effect of RA, if any, on the sensitivity of gastric cancer cells SC-M1 to LAK activity, with the goal of determining in vitro whether RA and IL-2 or LAK cells are effective for combination biological therapy. The results revealed that RA decreased the susceptibility of gastric cancer SC-M1 cells to LAK lysis. The underlying mechanism was in part caused by the effect of RA on cell cycle regulation, and the prevailing notions, such as the binding of effector cells to target cells, the expression of ICAM molecules or major histocompatibility

*Received 15 April 1996*
*Revised 8 July 1996*
*Accepted 19 September 1996*

*Correspondence to:* TY Chao, Division of Hematology/Oncology, Department of Medicine, Tri-Service General Hospital, No. 8, Ding-Chow Road, Section 3, Taipei, Taiwan, Republic of China complex (MHC) class I molecules and the alteration of RARβ, are not involved. These results may also advise caution as to the suitability of RA with LAK cells or IL-2 biotherapy in certain cancers, especially in the light of the most recent report describing the combination of β-carotene and vitamin A as having no benefit and possibly an adverse effect on lung cancer (Omenn et al, 1996).

### MATERIALS AND METHODS

### Target cells

Three cancer cell lines, including gastric cancer SC-M1, promyelocytic leukaemia HL-60 and hepatic cancer Hep 3B, were used as target cells in the cytotoxicity assay. Tumour cells were maintained in RPMI-1640 medium with 10% fetal calf serum (FCS) (Gibco, Grand Island, NY, USA).

### Recombinant IL-2

IL-2 was kindly provided by Cetus Oncology Corperation (Emeryville, CA, USA).

### Generation of LAK killer cell activity

LAK cell activity was generated as described previously (Chao et al, 1990, 1995*a*). Briefly, peripheral blood mononuclear cells (PBMCs) were obtained from normal healthy volunteers and incubated at 37°C in complete medium containing 3000 IU ml$^{-1}$ IL-2, under a moist atmosphere with 5% carbon dioxide in culture flasks at a cell concentration of 2–3 × 10$^{6}$ ml$^{-1}$. Complete medium contained RPMI-1640 with 10% FCS, 0.3 mg ml$^{-1}$ L-glutamine, 100 μg ml$^{-1}$ streptomycin and 100 U ml$^{-1}$ penicillin. Activated killer cells were harvested after culture for 4 days and washed twice with RPMI-1640. The viable cells were counted and resuspended in RPMI-1640 containing 10% FCS for the standard 4-h $^{51}$Cr-release assay (Chao et al, 1990, 1995*a*), and for surface marker studies with flow cytometry (Chao et al, 1995*b*).

### Co-culturing cancer cells with RA

RA (β-all-*trans*, Sigma, St Louis, MO, USA) was dissolved in a small amount of dimethyl sulphoxide (Sigma) to make a stock solution of 0.01 M. This solution was used at a final concentration ranging from 0.001–10 μM in complete medium. RA was added to the culture medium at the beginning of the culture. After incubation for various periods of time, tumour cells were harvested, washed and used as target cells in the standard 4-h $^{51}$Cr-release assay. Preliminary experiments showed dimethyl sulphoxide alone at the concentrations used had no effect on the susceptibility of cancer cells to LAK cytotoxicity.

### In vitro cytotoxicity assay

The standard 4-h $^{51}$Cr-release microcytotoxicity assay was performed as described (Chao et al, 1990, 1995*a*). Each cytotoxicity assay was performed in triplicate and the results were expressed as lytic units. To ensure the validity of the assay data, the maximum spontaneous release of $^{51}$Cr of target cells in this study was limited to 12%. There was no spontaneous cytotoxicity of RA on SC-M1 cells in our experiments. One lytic unit was defined as the number of effector cells required to cause 30% specific $^{51}$Cr release from 10$^{4}$ target cells and was expressed as lytic unit 10$^{-7}$ effector cells.

### Flow cytometric analysis of cell surface molecules on SC-M1 cells

Cell surface molecules on SC-M1 cells were examined by flow cytometry after immunofluorescence staining. Tumour cells cultured for 6 days, in the presence or absence of RA, were harvested, washed twice and resuspended in RPMI-1640. The cell concentrations were adjusted to 1 × 10$^{6}$ ml$^{-1}$ and incubated with mouse anti-human monoclonal antibodies against *HLA* class I and II antigens (Dako, Carpinteria, CA, USA), ICAM-1 and -2 (Bender, Vienna, Austria) or lymphocyte function antigen-3 (LFA-3) (Serotec, Oxford, UK), for 30 min at 4°C and then washed with phosphate-buffered saline (PBS) three times. These cells were then incubated with goat anti-mouse antibodies conjugated with fluorescein isothiocyanate for 30 min at 4°C. After three washes in PBS containing 0.05% Tween 20, cells stained in indirect immunofluorescence were resuspended in 200 μl of sheath fluid without azide and analysed in a flow cytometer (FACScan, Becton-Dickinson).

### Cell cycle analysis

Cell cycle analysis was performed by using flow cytometry as previously described (Shyu et al, 1995). Briefly, cells in logarithmic growth were labelled with 5 mM bromodeoxyuridine for 20 min. Cells were then harvested and fixed in 70% ethanol. Nuclei were prepared by incubating cells in 0.04% pepsin, 0.1 N hydrochloric acid for 20 min at room temperature, followed by the addition of 2 N hydrochloric acid at 37°C for 30 min. Following neutralization with 0.1 M sodium borate, nuclei were stained with anti-bromodeoxyuridine monoclonal antibody (Becton-Dickinson) at room temperature for 30 min and fluorescein-labelled goat anti-mouse antibody (1:50) (Sigma) for 30 min. Nuclei were then stained with 10 μg ml$^{-1}$ propidium iodide, 5 mg ml$^{-1}$ RNAase overnight at 4°C and analysed by FACScan. The fractions of cells in $G_0/G_1$, S and $G_2/M$ phases were analysed using the LYSYS program.

### Binding assay

Target cells (1 × 10$^{5}$) and LAK cells (5 × 10$^{5}$) are mixed in 0.2 ml of complete medium in an Eppendorf tube and incubated for 5 min at 37°C in a humidified 5% carbon dioxide atmosphere. The tubes were then centrifuged for 30 s, after which the pellet was pipetted gently three times and resuspended and incubated for another 5 min at 37°C in a humidified 5% carbon dioxide atmosphere. The percentage binding of effector cells to target cells was determined by counting at least 200 target cells.

### RNA preparation and Northern blot analysis

Poly (A)$^{+}$ RNA preparation and Northern blot analysis were performed as previously described (Shyu et al, 1995). Cells were lysed in buffer containing 0.2 M sodium chloride, 0.2 M Tris-HCl, pH 7.5, 1.5 mM magnesium chloride, 2% sodium dodecyl sulphate (SDS), 200 μg ml$^{-1}$ protease K and 50 μM aurintricarboxylic acid and incubated at 45°C for 2 h. Cell lysates were then incubated

© Cancer Research Campaign 1997

with oligo-dT cellulose (Boehringer Mannhiem) in the same buffer containing 0.5 M sodium chloride at room temperature for 1 h on a rotary shaker. After washing, RNA was eluted with 0.01 M Tris-HCl, pH 7.5. RNA was then fractionated on a 1.1% agarose, 1.1% formaldehyde gel in 5 mM NaOAc, 1 mM EDTA, 20 mM 3-[*N*-morpholino] propanesulphonic acid, pH 7.0, and transferred to a nylon membrane by capillary blotting in 20 × saline sodium citrate (SSC) (3 M sodium chloride, 0.3 M sodium citrate, pH 7.0). Blots were UV-fixed prehybridized and hybridized at 42°C in buffer containing 50% (v/v) formamide, 5 × SSC, 2% (w/v) blocking reagent, 0.1% *N*-lauroylsarcosine and 0.2 (w/v) SDS. The membranes were washed with 2 × SSC containing 0.1% SDS and then washed with 0.1 × SSC containing 0.1% SDS at 68°C for 30 min. Specific hybridization was detected by a DIG luminescent detection kit using lumigen-PPD as the substrate and was recorded using Kodak XAR-5 film at room temperature. To prepare membranes for rehybridization, membranes were incubated with 0.5% Tris-HCl, pH 8.0, 1% SDS and 50% formamide at 68°C for 1 h and then prehybridized as described above.

cDNA probes encoding *Drosophila* actin, mouse RARα, human RARβ and human RARγ were prepared as previously described (Shyu et al, 1995). The 1.7-kb cyclin A cDNA probe and the 1.4-kb cyclin B cDNA probe were kindly provided by Dr C Brechet and Dr T Hunter. The 1.25-kb GAPDH cDNA was provided by Dr R Wu. cDNAs were labelled with digoxigenin using a DNA labelling kit. The relative levels of RARα (3.5 and 2.9 kb), RARβ (3.4 and 3.1 kb) and RARγ (3.2 and 3.0 kb) were normalized to the level of β-actin mRNA from the same nylon membrane. Normalizations of the levels of cyclin A and cyclin B1 were not performed because RA also induced a decreased expression of GAPDH mRNA.

### Statistical analysis

The mean and standard errors of the mean (s.e.m.) of the data were calculated. Statistical analyses were performed by Student's *t*-test for individual and for paired samples.

### RESULTS

### Effect on susceptibilities of SC-M1 cells to LAK cytotoxicity by various concentrations of RA

The possible change in susceptibility of SC-M1 cells to LAK cytotoxicity as induced by RA was initially determined after SC-M1 cells were co-cultured with RA at various concentrations for 6 days. As shown in Figure 1, susceptibility of SC-M1 cells to LAK lysis generated from culturing PBMCs of healthy donors with IL-2 was reduced, and the reduction was RA dose dependent at concentrations ranging from 0.001–10 μM that were examined. A significant decrease in LAK cytotoxicity was detected at RA concentrations of 0.1 μM ($P$=0.0015) and 10 μM ($P$=0.0002). The RA concentration of 5 μM was selected for further experiments.

RA at 5 μM was added to SC-M1 culture medium and SC-M1 cells were harvested for LAK cytotoxicity assay after 2, 6 and 10 days of incubation. As shown in Figure 2, the resistance of RA-treated SC-M1 cells to LAK lysis was time dependent. To ascertain the validity of RA-induced resistance to LAK cytotoxicity, two additional cancer cell lines, a leukaemia cell line HL-60 and a hepatic cancer cell line Hep 3B, were used as target cells in kinetic studies. To determine the time required for the development of a significant RA-induced resistance, tumour cells were cultured with RA at 5 μM for 1, 2, 6 and 10 days before testing their sensitivity to LAK lysis. Figure 3 shows that the RA-induced resistance to LAK cytotoxicity was also detected in both HL-60 and Hep 3B cell lines. Although a difference in the sensitivity to LAK lysis was detected between SC-M1 cells and HL-60 and Hep 3B cells, the kinetics of resistance development was similar. A significant resistance to

**Figure 1** The effect of various concentrations of RA, i.e. dose dependent, on susceptibility of SC-M1 tumour cells to LAK lysis. SC-M1 cells were incubated with RA at the concentrations indicated for 6 days before being used as target cells in a cytotoxicity assay. The LAK activity expressed as lytic units was generated by co-culturing PBMCs of normal donors with 3000 IU ml$^{-1}$ IL-2 for 4 days and was measured by standard 4-h $^{51}$Cr-release assay. The results are expressed as means ± s.e.m. (bars) from triplicate assays

**Figure 2** The effect of the length of RA treatment on sensitivity of SC-M1 tumour cells to LAK lysis. Untreated SC-M1 cells ■ and SC-M1 cells treated with RA at 5 μM ▣ for various periods of time in culture were used as target cells in a LAK cytotoxicity assay. The results are expressed as means ± s.e.m. (bars) from triplicate assays

© Cancer Research Campaign 1997

**Figure 3** Induction of resistance by RA to LAK cytotoxicity in gastric (□; SC-M1), promyelocytic leukaemia (◆; HL-60) and hepatic (■; Hep 3B) cancer cell lines. Cancer cells were incubated with RA at 5 μM for various periods of time, as indicated, before being used as target cells in a LAK cytotoxicity assay. The results represent means ± s.e.m. (bars) from triplicate assays

**Figure 4** Expression of cyclin mRNA in SC-M1 cells treated with RA at 5 μM for 12 and 96 h. Poly(A)+ RNA (3 μg per lane) was hybridized with digoxigenin-labelled cDNA for cyclin A and cyclin B1. Probes were removed and membranes were rehydridized with a GAPDH probe as shown on the lower section of each panel

LAK lysis occurred when target cells were cultured with RA for as little as 2 days in culture and reached a maximum plateau after culture for 6 days (data from culture of RA for 10 days not shown).

### Cell cycle phase distribution and cyclins analysis

The effect of RA on the cell cycle phase distribution was analysed by flow cytometry. As shown in Table 1, treatment of non-synchronized SC-M1 cells with RA at the concentrations of 0.01 μM and 1 μM resulted in a significant increase in the fractions of cells in $G_0/G_1$ phase ($P$<0.05 and $P$<0.01 respectively) and a significant decrease in the fraction of cells in S phase ($P$<0.01 at 1 μM RA). Also noted is that the changes were in a RA dose dependent manner.

Additional experiments were performed to examine the expression of cyclin A and cyclin B1 mRNA in SC-M1 cells treated with RA. Figure 4 shows that a decrease in the expression of cyclin A mRNA (2.8 and 2.0 kb) and cyclin B1 mRNA (1.7 kb) was detected in SC-M1 cells after co-culture with RA (5 μM) for 96 h, but not at 12 h. Results also show that a significant decrease in cyclin A and cyclin B1 mRNA was detected after SC-M1 cells were cultured with RA for 24 h, and that the decrease in cyclins A and B1 by RA was in both a dose- and time-dependent manner (data not shown).

### Formation of conjugates between effector LAK and target tumour cells

To determine the effect of RA treatment on the recognition of tumour cells by LAK cells, RA-treated and untreated tumour cells were compared in a direct binding assay in which conjugate formation between effector LAK cells and target tumour cells, SC-M1 and Hep 3B, was examined directly by a microscope. The treatment of SC-M1 cells and Hep 3B cells with RA at 5 μM for 6 days reduced their susceptibilities to lysis by LAK cells as shown previously (Figure 3), yet no significant difference in the frequency of LAK cells forming the conjugates with RA-treated SC-M1 cells or Hep 3B cells was discernible (Table 2). The results suggested that the reduced LAK lysis was not caused by any decrease in binding or contact between effectors and target cells.

**Table 1** Effect of RA on the cell cycle distribution of SC-M1 cells[a]

| | Percentage of cells in cell cycle phase | | |
|---|---|---|---|
| **Treatments** | $G_0/G_1$ | S | $G_2/M$ |
| SC-M1 | 49.4 ± 0.1[b] | 27.7 ± 4.7 | 22.1 ± 4.5 |
| SC-M1 + RA (0.01 μM) | 56.9 ± 2.0* | 23.5 ± 4.0 | 18.4 ± 1.7 |
| SC-M1 + RA (1 μM) | 67.8 ± 2.1** | 9.4 ± 2.1** | 21.9 ± 4.5 |

[a]After culture with RA at the concentrations shown for 6 days, distribution of cell cycle phase was analysed by flow cytometry. [b]The number represents mean ± s.e.m. of three experiments. *$P$ < 0.05, **$P$ < 0.01, for SC-M1 vs SC-M1 + RA.

**Table 2** Comparison of conjugate formation between effector LAK cells and target SC-M1 cells and Hep 3B cells with and without treatment with RA

| Target cell | Conjugate/total target cells (%) |
|---|---|
| SC-M1 | 48.7 ± 2.5[a,b] |
| SC-M1 + RA[c] | 50.3 ± 0.7 |
| Hep 3B | 23.8 ± 2.1 |
| Hep 3B + RA[c] | 24.3 ± 1.9 |

[a]The numbers are calculated from counting 200 target cells and are expressed as mean ± s.e.m. from three experiments. [b]All $P$-values are > 0.05 for SC-M1 vs SC-M1 + RA. [c]Cultured with RA at 5 μM for 6 days.

© Cancer Research Campaign 1997

### Northern blot analysis of RAR

Because a significant resistance to LAK in SC-M1 cells was detected after treatment with RA for 2 days, changes in steady-state levels of RAR mRNA in SC-M1 cells treated with RA for 2 days were analysed. Following exposure to RA for 2 days, steady-state levels of two RARα mRNA transcripts (3.5 and 2.9 kb) were increased in SC-M1 cells in a RA dose-dependent manner (Figure 5, top). The 4.8-kb band was derived from cross-hybridization with 28S ribosomal RNA and was not regulated by RA. No detectable level of RARβ mRNA was observed in SC-M1 cells (Figure 5, middle). The expression of two RARγ mRNA transcripts (3.2 and 3.0 kb) remained unchanged (Figure 5, bottom).

**Table 3** Flow cytometric analysis of the expression of HLA, ICAM and LFA molecules on SC-M1 cells

| Molecules examined | SC-M1 | SC-M1 + RA[a] |
|---|---|---|
| HLA class I | 100.7 ± 6.2[b,c] | 93.1 ± 6.0[b,c] |
| HLA class II | 1.5 ± 0.2 | 1.3 ± 1.1 |
| ICAM-1 | 101.6 ± 13.6 | 101.2 ± 13.9 |
| ICAM-2 | 0.7 ± 0.6 | 1.3 ± 1.3 |
| LFA-3 | 70.9 ± 7.6 | 66.4 ± 5.0 |

[a]The SC-M1 cells were cultured with RA (5 μM) for 6 days. [b]The numbers represent fluorescence intensity and are expressed as mean ± s.e.m. from five experiments. [c]All *P*-values are >0.05 for SC-M1 vs SC-M1 + RA.

### Expression of cell surface and adhesion molecules

Cytotoxicity mediated by LAK cells involves adhesive interactions between LFA-1 (CD 11a/CD 18) and CD 2 on immune effectors and ICAM-1, -2 (CD 54 and CD 102) as well as LFA-3 (CD 58) on tumour cell targets (Robertson et al, 1990; Foreman et al, 1993). Although LAK lysis is MHC unrestricted, the expression of MHC molecules may play a regulatory role (De Fries and Golub, 1988). These surface and adhesion molecules and their ligands are not only important in effector to target cell adhesion, but also play a significant role in effector activation (Galandrini et al, 1992). Therefore, whether expression of ICAM-1 and -2, LFA-3 and HLA class I and II molecules were different on SC-M1 cells from those on RA-treated SC-M1 cells was examined by flow cytometry. As shown in Table 3, SC-M1 cells expressed HLA class I, ICAM-1 and LFA-3, but not HLA class II or ICAM-2 molecules. These results indicated that the expression of these adhesion molecules was similar in SC-M1 tumour cells with and without treatment with RA.

**Figure 5** Expression of RAR mRNA in SC-M1 cells treated with various concentrations of RA for 2 days. Poly (A)+ RNA (10 μg per lane) was hybridized with digoxigenin-labelled cDNA for RARα, RARβ and RARγ. Probes were removed and membranes were rehybridized with β-actin probe as shown on the lower section of each panel

### DISCUSSION

Retinoids are becoming increasingly useful therapeutic agents for some neoplasms, such as premalignant and malignant diseases affecting the skin, head and neck, lung, bladder, uterine cervix and bone marrow (Smith et al, 1992). Retinoids are known to exert a variety of direct effects on tumour cells, including the induction of differentiation and apoptosis (Martin et al, 1990) and the inhibition of cell proliferation (Lotan et al, 1990). Morphological changes, alterations in expression of cell surface molecules, notably adhesion molecules, and a number of metabolic and enzymatic changes have been reported (Lotan et al, 1990; Triozzi et al, 1992; Bouillon and Audette, 1994). Thus, the effects of retinoids on tumour cells are numerous and any of these retinoid-induced changes, alone or in combination, may contribute to the reduced sensitivity to LAK-mediated cytolysis that we have described.

In the present study, we have reported that susceptibility of gastric cancer, promyelocytic leukaemia and hepatic cancer cell lines to LAK lysis was greatly reduced by RA. Similar phenomena of RA-inducible resistance to LAK cytotoxicity have also been reported. The exposure of HL-60 cells to RA, interferon-α, interferon-β or interferon-γ has produced an increased protection from LAK cytolysis (Triozzi et al, 1992). Interferon-γ has also been reported to induce protection of a variety of cancer cell lines, e.g. renal cell cancer, melanoma, sarcoma and lymphoma, from LAK cytolysis (De Fries and Golub, 1988). Additionally reported is that phorbol 12-myristate 13-acetate, a differentiation enhancer, induced melanoma cells to generate resistance to LAK cells, while undergoing growth inhibition and neuron-like differentiation (Correale et al, 1992).

Although interferon has been implicated as playing a role, the exact mechanisms underlying RA-enhanced resistance of cancer cells to LAK cell activity remain unknown. Any alteration of cancer cells, which may interfere with any of the stages of cell-mediated

*© Cancer Research Campaign 1997*

cytotoxicity, i.e. recognition, triggering, programming and lysis, could be responsible for the decreased susceptibility of LAK-mediated cytotoxicity (Apasov et al, 1993; Henkart, 1994). These may include a reduction or masking of relevant recognition structures, a reduced ability to trigger the release of cytotoxic mediators, a decrease in transduction of cytotoxic signals and/or an increase in cellular repairing.

The recognition structures or factors on the target cancer cell surface that mediate LAK cytolysis remain largely unknown. Cellular adhesion molecules such as ICAM-1, ICAM-2, LFA-3 and MHC class I and II antigens have been proposed by some investigators as playing an auxiliary but critical role in the initial contact between immune effectors and target tumour cells, but the proposition is not universally accepted (De Fries and Golub, 1988; Robertson et al, 1990; Correale et al, 1992; Galandini et al, 1992; Triozzi et al, 1992; Fady et al, 1993; Foreman et al, 1993; Henkart, 1994; Katsanis et al, 1994). In agreement with the majority of these reports, our results indicate that the expression of ICAM-1, ICAM-2, LFA-3 and MHC class I and II molecules are not associated with the RA-induced susceptibility of SC-M1 cells to LAK lysis. Our other data obtained from direct examination of immune effector–tumour cells conjugate formation also reveal that the recognition and contact between cancer cells and effectors are not involved. We have shown the presence of RARβ and RARγ mRNAs and the absence of RARβ mRNA in SC-M1 cells (Shyu et al, 1995). In the present study, we have further demonstrated that the expression of RARα mRNA in SC-M1 cells was significantly increased by co-culturing with RA, and that the expression of RARβ and RARγ mRNA was not affected. In fact, RARβ and RARγ mRNA in SC-M1 cells remain unchanged throughout the duration of co-culturing with RA for a period of 2 years (SY Jiang, unpublished data). It is likely that RARα may be involved in the altered susceptibility of SC-M1 cells to LAK cell lysis, whereas RARβ and RARγ would not play any role. RXRs were not examined in this study, as we have shown that SC-M1 cells express no RXRα, while the limited amount of RXRα and RXRβ expressed as not correlated with RA sensitivity.

Cellular proliferation follows an orderly progression through the cell cycle. Of interest to note is that incubation of SC-M1 cells with RA results in an increase in $G_0/G_1$ phase and a decrease in S phase, as analysed by cell cycle distribution. Morphologically, culture with RA produces 'enlarged and flattened' SC-M1 cells (Shyu et al, 1995). Neither a reduced viability of cells nor evidence of apoptosis, such as nuclear fragmentation or chromatin condensation, has been observed in RA-treated SC-M1 cells (Shyu et al, 1995). Cyclin analysis has revealed that cyclin A and cyclin B1, the commonly regarded regulators of the transition to mitosis (Cordon-Cardo, 1995), are greatly reduced. Taken together, these results suggest that RA exerts a cytostatic effect on SC-M1 cells. Furthermore, LAK-mediated cytolysis requires an active participation from the target cells that are to be metabolically active (Zychlinsky et al, 1991). Therefore, the RA-induced cytostatic effect in SC-M1 cells may derive from such a mechanism that eventually results in a reduction of sensitivity to LAK cytotoxicity as observed. Similar observations have been reported in breast cancer cells and leukaemia cells in which susceptiblity to LAK cytotoxicities increases when target cancer cells become proliferative in response to the stimulation of oestradiol and granulocyte–monocyte colony-stimulating factor respectively (Albertini et al, 1992; Teichmann et al, 1992). One report has shown that LAK cells are able to bind target cells independently of the cell cycle phase in Chang cell lines as measured by cytometric and morphological parameters (Nano et al, 1995). However, binding between LAK cells and their targets is not equal to lysis. Our results in this study have also demonstrated that the binding between LAK cells and targets was unchanged by RA modification of SC-M1 cells.

In summary, the present study has indicated that culture of SC-M1 gastric cancer cells with RA has produced a decrease in their susceptibilities to LAK-mediated cytotoxicity. The prevailing notions, such as a reduced binding between immune effectors and target tumour cells, a decreased expression of ICAM and MHC class I molecules and an alteration of RARβ, appear not to be involved. The underlying mechanism is caused in part by a cytostatic effect of RA on target tumour cells. The phenomenon seen applies to a range of cell lines and further study would be advisable in those tumours for which retinoic acid therapy and IL-2 are contemplated.

### ACKNOWLEDGEMENTS

This work was supported by a grant (NSC 85-2331-B-016-062) awarded by the National Science Council of the Republic of China (to T-Y C). The authors would like to thank Dr Dah-Shyong Yu and Mr Kuo-Cheu Yueh for their assistance in flow cytometric analysis, and Ms J Ogledzinski for secretarial assistance.

### REFERENCES

Albertini MR, Gibson DF, Robinson SP, Howard SP, Tans KJ, Lindstrom MJ, Robinson RR, Tormey DC, Jordan VC and Sondel PM (1992) Influence of estradiol and tamoxifen on susceptibility of human breast cancer cell lines to lysis by lymphokine-activated killer cells. *J Immunother* **11**: 30–39

Apasov S, Redegeld F and Sitkovsky M (1993) Cell-mediated cytotoxicity: contact and secreted factors. *Curr Opin Immunol* **5**: 404–410

Athanassiades T (1981) Adjuvant effect of vitamin A palmiate and analogs on cell-mediated immunity. *J Natl Cancer Inst* **67**: 1153–1156

Bollag W and Peck R (1993) Modulation of growth and differentiation by combined retinoids and cytokines in cancer. In *Retinoids in Oncology*, Hong WK and Lotan R (eds), pp. 89–108. Marcel Dekker:New York

Bouillon M and Audette M (1994) Retinoic acid-stimulated intercellular adhesion molecule-1 expression on SK-N-SH cells: calcium/calmodulin-dependent pathway. *Cancer Res* **54**: 4144–4149

Butler WB and Fontana JA (1992) Responses to retinoic acid of tamoxifen-sensitive and -resistant subline of human breast cancer cell line MCF-7. *Cancer Res* **52**: 6164–6167

Chao TY, Ohnishi H and Chu TM (1990) Indirect inhibition of generation of murine lymphokine-activated killer cell activity in splenocyte cultures by interferon-gamma. *Immunology* **70**: 116–120

Chao TY, Ting CS, Yeh MY, Chang JY, Wang CC and Chu TM (1995*a*) Effects of indomethacin on lymphokine-activated killer cell activities in cancer patients. *Tumor Biol* **16**: 230–242

Chao TY, Huang WS and Yeh MY (1995*b*) Generation of lymphokine-activated killer (LAK) cell activity from malignant peritoneal effusions. *Proc Natl Sci Council ROC Part B* **19**: 92–98

Cohen PS, Letterio JL, Gaetano C, Chan J, Matsumoto K, Sporn MB and Thiele CJ (1995) Induction of transforming growth factor $\beta_1$ and its receptors during all-*trans*-retinoic acid (RA) treatment of RA-responsive human neuroblastoma cell lines. *Cancer Res* **55**: 2380–2386

Cordon-Cardo C (1995) Mutation of cell cycle regulators: biological and clinical implications for human neoplasia. *Am J Pathol* **147**: 545–560

Correale P, Procopio A, Celio L, Caraglia M, Cenua G, Coppola V, Pepe S, Normanno N, Vecchio I, Palmieri G, Montagnani S, Tagliaferri P and Bianco AR (1992) Phorbol 12-myristate 13-acetate induces resistance of human melanoma cell to natural-killer- and lymphokine-activated-killer-mediated cytotoxicity. *Cancer Immunol Immunother* **34**: 272–278

© Cancer Research Campaign 1997

De Fries RU and Golub SH (1988) Characteristics and mechanisms of IFN-γ induced protection of human tumor cells from lysis by lymphokine-activated killer cells. *J Immunol* **140**: 3686–3693

Degos L (1992) Retinoic acid in acute promyelocytic leukemia: a model for differentiation therapy. *Curr Opin Oncol* **4**: 45–52

Dillehay D, Walia A and Lamon E (1988) Effects of retinoids on macrophage function and IL-1 activity. *J Leuk Biol* **44**: 353–360

Dmitrovsky E, Markman M and Marks PA (1990) Clinical use of differentiating agents in cancer therapy. In *Cancer Chemotherapy and Biologic Response Modifiers Annual 11*, Pinedo HM, Chabner GA and Longo DL (eds), pp. 303–320. Elsevier: Amsterdam

Fady C, Gardner A, Gera JF and Lichtenstein A (1993) Interferon-γ-induced increased sensitivity of HER2/neu-overexpressing tumor cells to lymphokine-activated killer cell lysis: importance of ICAM-1 in binding and post-binding events. *Cancer Immunol Immunother* **37**: 329–336

Fegan C, Bailey-Wood R, Coleman S, Phillips SA, Neale L, Hoy T and Whittaker JA (1995) All *trans* retinoic acid enhances human LAK activity. *Eur J Hematol* **54**: 95–100

Foreman NK, Rill DR, Coustan-Smith E, Douglass EC and Brenner MK (1993) Mechanisms of selective killing of neuroblastoma cells by natural killer cells and lymphokine-activated killer cells. Potential for residual disease eradication. *Br J Cancer* **67**: 933–938

Galandrini R, Albi N, Zarcone D, Grossi CE and Velardi A (1992) Adhesion molecule-mediated signals regulate major histocompatibility complex-unrestricted and CD3/T cell receptor-triggered cytotoxicity. *Eur J Immunol* **22**: 2047–2053

Henkart PA (1994) Lymphocyte-mediated cytotoxicity: two pathways and multiple effector molecules. *Immunity* **1**: 343–346

Huang ME, Ye YC, Chen SR, Chai JR, Lu JX, Zhoa L, Gu LJ and Wang ZY (1988) Use of all-*trans* retinoic acid in the treatment of acute promyelocytic leukemia. *Blood* **72**: 567–572

Katsanis E, Bausero MA, Xu H, Orchard PJ, Xu Z, Mcivor RS, Brian AA and Blazar BR (1994) Transfection of the mouse ICAM-1 gene into murine neuroblastoma enhances susceptibility to lysis, reduced in vivo tumorigenicity and decreases ICAM-2-dependent killing. *Cancer Immunol Immunother* **38**: 135–141

Lin TH and Chu TM (1990) Enhancement of murine lymphokine-activated killer cell activity by retinoic acid. *Cancer Res* **50**: 3013–3018

Lotan R, Lotan D and Sacks PG (1990) Inhibition of tumour cell growth by retinoids. *Methods Enzymol* **190**: 100–110

Love JM and Gudas LJ (1994) Vitamin A, differentiation and cancer. *Curr Opin Cell Biol* **6**: 825–831

Martin S, Bradley J and Cotter T (1990) HL-60 cells induced to differentiate towards neutrophils subsequently die via apoptosis. *Clin Exp Immunol* **79**: 448–453

Nano R, Barni S, Capelli E, Prosperi E, Lavezzi L and Salvucci O (1995) DNA-protein cell content of lymphokine-activated killer (LAK) and target cells in coculture. *Anticancer Res* **15**: 751–754

Omenn GS, Goodman GE, Thornquist MD, Balmes J, Cullen MR, Glass A, Keogh JP, Meyskens FL Jr, Valanis B, Williams JH Jr, Barnhart S and Hammar S (1996) Effects of a combination of beta carotene and vitamin A on lung cancer and cardiovascular disease. *N Engl J Med* **334**: 1105–1155

Palumbo A, Battaglio S, Napoli P, Bruno B, Omede P, Boccadoro M and Pileri A (1995) Retinoic acid inhibits the growth of human myeloma cells in vitro. *Br J Hematol* **89**: 555–560

Robertson MJ, Caligiuri MA, Manley TJ, Levine H and Ritz J (1990) Human natural killer cell adhesion molecules. Differential expression after activation and participation in cytolysis. *J Immunol* **145**: 3194–3201

Shyu RY, Jiang SY, Huang SL, Chang TC, Wu KL, Roffler SR and Yeh MY (1995) Growth retardation by all-*trans*-retinoic acid and retinoic acid receptor messenger ribonucleic acids expression in gastric cancer cells. *Eur J Cancer* **31A**: 237–243

Smith MA, Parkinson DR, Cheson BD and Friedman MA (1992) Retinoids in cancer therapy. *J Clin Oncol* **10**: 839–864

Strickland S and Sawey MJ (1980) Studies on the effect of retinoids on the differentiation of teratocarcinoma stem cells in vitro and in vivo. *Dev Biol* **78**: 76–85

Teichmann JV, Ludwig WD and Thiel E (1992) GM-CSF-mediated proliferation induction improves the susceptibility of leukemia cells to lymphokine-activated killer cells. *Int J Hematol* **55**: 255–264

Triozzi PL, Eicher DM, Smoot J and Rinehart JJ (1992) Modulation of leukemic cell sensitivity to lymphokine-activated killer cytolysis: role of intercellular adhesion molecule-1. *Exp Hematol* **20**: 1072–1076

Villa ML, Ferrario E, Trabattoni D, De Palo G, Magni A, Veronesi U and Clerici E (1993) Retinoids, breast cancer and NK cells. *Br J Cancer* **68**: 845–850

Warrell RP Jr, Frankel SR, Miller JR. WH, Scheinberg DA, Itri LM, Hittelman WN, Vyas R, Andreeff M, Tafuri A, Jakubowski A, Gabrilove J, Gordon MS and Dmitrovsky E (1991) Differentiation therapy of acute promyelocytic leukemia with tretinoin (all-*trans*-retinoic acid). *N Engl J Med* **324**: 1385–1393

Warrell RP Jr, De The H, Wang ZY and Degos L (1993) Acute promyelocytic leukemia. *N Engl J Med* **329**: 177–189

Zou CP, Clifford JL, Xu XC, Sacks PG, Chambon P, Hong WK and Lotan R (1994) Modulation by retinoic acid (RA) of squamous cell differentiation, cellular RA-binding proteins, and nuclear RA receptors in human head and neck squamous cell carcinoma cell lines. *Cancer Res* **54**: 5479–5487

Zychlinsky A, Zheng LM, Liu C-Y and Young JD-E (1991) Cytolytic lymphocytes induce both apoptosis and necrosis in target cells. *J Immunol* **146**: 393–400

© Cancer Research Campaign 1997